# Activating transcription factor 3, glucolipid metabolism, and metabolic diseases

**DOI:** 10.1093/jmcb/mjac067

**Published:** 2022-12-06

**Authors:** Shuwei Hu, Xiaojie Zhao, Rui Li, Chencheng Hu, Huijuan Wu, Jing Li, Yanqiao Zhang, Yanyong Xu

**Affiliations:** Department of Integrative Medical Sciences, Northeast Ohio Medical University, Rootstown, OH 44272, USA; Department of Pathology, School of Basic Medical Sciences, Fudan University, Shanghai 200032, China; Department of Pathology, School of Basic Medical Sciences, Fudan University, Shanghai 200032, China; Department of Pathology, School of Basic Medical Sciences, Fudan University, Shanghai 200032, China; Department of Pathology, School of Basic Medical Sciences, Fudan University, Shanghai 200032, China; Department of Endocrinology, Beijing Chao-yang Hospital, Capital Medical University, Beijing 100020, China; Department of Integrative Medical Sciences, Northeast Ohio Medical University, Rootstown, OH 44272, USA; Key Laboratory of Metabolism and Molecular Medicine of the Ministry of Education, Department of Pathology of School of Basic Medical Sciences, Fudan University, Shanghai 200032, China; Frontier Innovation Center, School of Basic Medical Sciences, Fudan University, Shanghai 200032, China

**Keywords:** ATF3, glucolipid metabolism, metabolic organ, metabolic diseases

## Abstract

Lipids and glucose exert many essential physiological functions, such as providing raw materials or energy for cellular biosynthesis, regulating cell signal transduction, and maintaining a constant body temperature. Dysregulation of lipid and glucose metabolism can lead to glucolipid metabolic disorders linked to various metabolic diseases, such as obesity, diabetes, and cardiovascular disease. Therefore, intervention in glucolipid metabolism is a key therapeutic strategy for the treatment of metabolic diseases. Activating transcription factor 3 (ATF3) is a transcription factor that acts as a hub of the cellular adaptive-response network and plays a pivotal role in the regulation of inflammation, apoptosis, DNA repair, and oncogenesis. Emerging evidence has illustrated the vital roles of ATF3 in glucolipid metabolism. ATF3 inhibits intestinal lipid absorption, enhances hepatic triglyceride hydrolysis and fatty acid oxidation, promotes macrophage reverse cholesterol transport, and attenuates the progression of western diet-induced nonalcoholic fatty liver disease and atherosclerosis. In addition to its role in lipid metabolism, ATF3 has also been identified as an important regulator of glucose metabolism. Here, we summarize the recent advances in the understanding of ATF3, mainly focusing on its role in glucose and lipid metabolism and potential therapeutic implications.

## Graphical Abstract

**Graphical Abstract:**
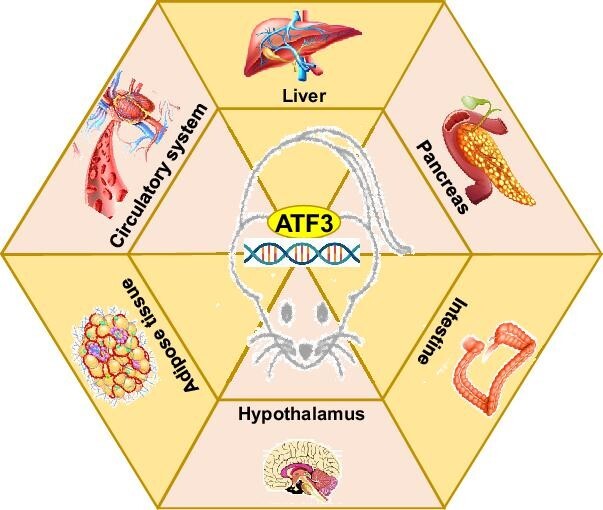

## Introduction

**Figure 1:**
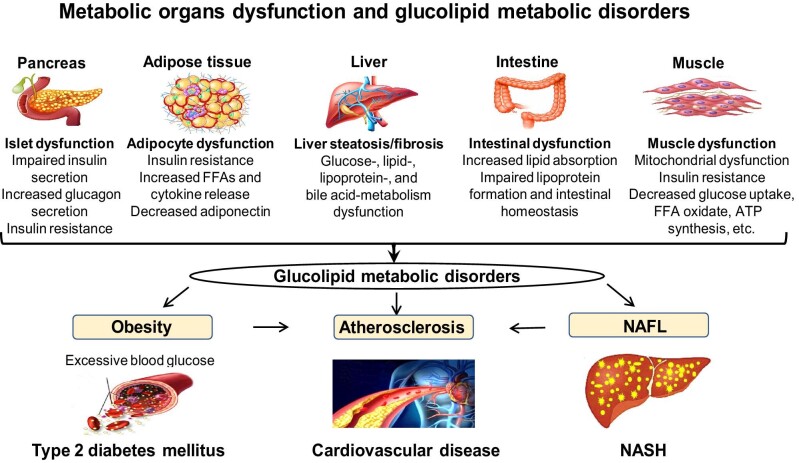
Metabolic organ dysfunction, glucolipid metabolic disorders, and metabolic diseases. To maintain metabolic homeostasis, the human body employs many physiological metabolic organs, such as the liver, adipose tissue, intestine, pancreas, muscle, etc. Dysregulation of lipid and glucose metabolism in these metabolic organs can lead to glucolipid metabolic disorders, leading to obesity, type 2 diabetes mellitus, NAFL, NASH, and CAD.

Glucolipid metabolic disorder, characterized by dysregulation of glucose and lipid metabolism in major metabolic organs or tissues, is one of the leading chronic disorders causing obesity, type 2 diabetes mellitus, nonalcoholic fatty liver disease (NAFLD), and atherosclerotic cardiovascular disease (CAD) (Figure 1; Chen et al., 2019; Brennan et al., 2021). Targeting glucose and/or lipid metabolism has become a promising therapeutic strategy for maintaining systemic homeostasis and preventing metabolic diseases in patients who live with glucolipid metabolic disorders. Intervention involving targets that regulate glucolipid metabolism with the aim of halting the progression of metabolic diseases has been extensively explored over the past decades. Among these targets, transcription factors are essential for maintaining metabolic homeostasis, which allows cells or an organ to respond to a variety of intracellular or extracellular signals, thus playing vital roles in glucolipid metabolism occurring in various metabolic organs or controlled by inter-organ communication (Priest and Tontonoz, 2019; Filipovic et al., 2021; Ke et al., 2021).

Activating transcription factor 3 (ATF3) is a member of the ATF/cyclic adenosine monophosphate (cAMP) response element-binding family that regulates gene transcription by binding to the consensus sequence TGACGTCA in target gene promoters (Montminy and Bilezikjian, 1987; Deutsch et al., 1988). Interestingly, ATF3 can also interact with other proteins via its basic leucine zipper domain and regulate cellular functions independently of its transcriptional activity (Chen et al., 1994). Humans and mice share 95% amino acid identity for ATF3. As a hub of the cellular adaptive-response network, ATF3 is expressed at relatively low levels in normal and quiescent cells, but its expression can be markedly upregulated by multiple extracellular and intracellular stress signals, such as endoplasmic reticulum stress, cytokines, chemokines, lipopolysaccharide (LPS), aberrant DNA damage, and oxidative injury (Zhao et al., 2016; Qian et al., 2017; Jeong, 2018). Increasing evidence indicates that ATF3 plays important roles in anti-inflammatory and immune responses, oncogenesis, and metabolic homeostasis (Jadhav and Zhang, 2017; Ku and Cheng, 2020). ATF3, on the other hand, is present in a wide variety of metabolic organs and tissues, such as the liver, adipose tissue, intestine, pancreas, and hypothalamus. Given that metabolic functions vary greatly among organs and tissues, ATF3 may display cell- or tissue-specific regulation of metabolic pathways in different tissues in systemic glucolipid metabolism. Here, we briefly review the recent discoveries regarding the roles of ATF3 in glucose and lipid metabolism and metabolic diseases.

## Roles of hepatic ATF3 in glucose and lipid metabolism

The liver is a key metabolic organ that governs glucolipid metabolism, and its dysfunction may cause NAFLD, type 2 diabetes mellitus, dyslipidemia, etc. The regulatory network of hepatic glucolipid metabolism is orchestrated by a delicate interplay of signaling molecules, intracellular signaling pathways, and transcription factors. Previous reports have indicated that ATF3 plays a protective role in the progression of high-fat diet-induced nonalcoholic fatty liver (NAFL) and nonalcoholic steatohepatitis (NASH) through inhibition of liver inflammation, hepatocellular apoptosis, hepatic stellate cell activation, and fibrosis (Table 1). However, little is known about the physiological functioning of ATF3 in hepatic glucolipid metabolism and metabolic disease or the underlying mechanisms. Until 2001, the role of hepatic ATF3 in gluconeogenesis was first reported by Allen-Jennings et al. (2001), who found that transgenic expression of ATF3 in the liver repressed hepatic expression of phosphoenolpyruvate carboxykinase (PEPCK) and fructose-1,6-bisphosphatase (FBP), two rate-limiting enzymes in gluconeogenesis. However, Kim et al. (2017) reported that injection of ATF3 siRNA into zucker diabetic fatty rats induced fatty acid oxidation, inhibited inflammatory responses, and improved glucose tolerance, and thus they proposed that ATF3 could be a target molecule linking hepatic steatosis to impaired glucose homeostasis. The latter study was performed in rats and used nongenetic approaches to knock down ATF3 expression globally, which has been challenged by the use of genetic mouse models.

**Table 1:** Pleiotropic role of ATF3 in liver disease.

Affected pathway	Disease model	Targets/pathway	References
Oxidative stress/inflammatory	Liver IRI, NAFLD, NASH,HCC	mTOR/p70S6K/HIF-1α, IRF2BP2, CRLS1, NUPR1, IL-6Rα, NRF2, p65	Kwon et al. (2015); Zhu et al. (2018); Nagahara et al. (2019); Fang et al. (2020); Tu et al. (2020); Bardallo et al. (2022); Dai et al. (2022)
Apoptosis	HCC, HepG2, NAFLD/NASH	LINC00238/TMEM106, HNF4α, BIM	Li et al. (2019); Jiang et al. (2021); Xu et al. (2021a); Kim et al. (2022)
Fibrosis	CCl_4_-induced liver fibrosis, NASH	TGF-β, α-SMA, lnc-SCARNA10, caspase-3	Shi et al. (2020); Xu et al. (2021a)

IRI, ischemia/reperfusion injury; HCC, hepatocellular carcinoma.

**Figure 2:**
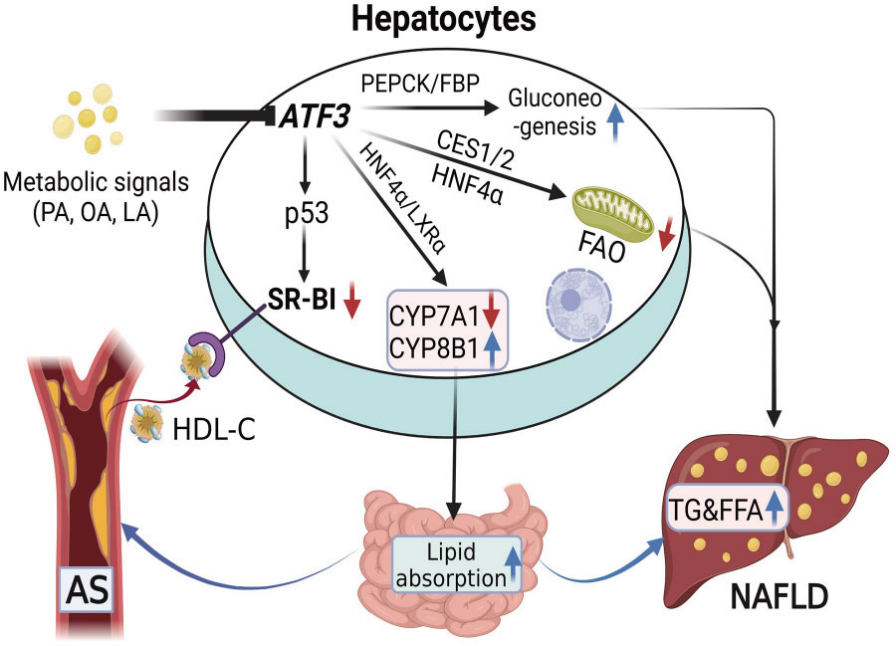
Hepatocyte ATF3 regulates lipid and glucose metabolism and protects against metabolic disorders. ATF3 improves lipid and glucose metabolism by controlling triglyceride (TG) metabolism, bile acid metabolism, HDL metabolism, and gluoneogenesis in hepatocytes by regulating the transcription of rate-limiting enzymes such as CES1/2, CYP7A1/8B1, SR-BI, PEPCK, etc. Metabolic signals such as PA, OA, and LA repress ATF3. AS, atherosclerosis; HDL-C, high-density lipoprotein cholesterol.

Very recently, two studies have shown that hepatic ATF3 expression is markedly reduced in patients with NAFLD and in mouse models of diabetes or obesity (Xu et al., 2021a). These results are in agreement with an earlier study showing that ATF3 mRNA expression was reduced in the livers of individuals with obesity (Cheng et al., 2019). In addition, the authors found that hepatocyte ATF3 expression can be reduced in response to metabolic signals. More importantly, they found newly uncovered mechanisms of ATF3 action in the transcriptional regulation of hepatic lipid metabolism, thus revealing an unanticipated protective role of ATF3 in the development and progression of NAFLD and atherosclerosis (Xu et al., 2021a, b). Adeno-associated virus-mediated ATF3 overexpression in hepatocytes prevented diet-induced steatohepatitis in C57BL/6 mice and reversed steatohepatitis in db/db mice. Conversely, global or hepatocyte-specific loss of ATF3 aggravated diet-induced steatohepatitis. Mechanistically, hepatocyte HNF4α was shown to interact with ATF3 and mediate the effects of ATF3 on inducing hepatic lipolysis and fatty acid oxidation via transcriptional regulation of carboxylesterase 1 and carboxylesterase 2 expression. In addition, the authors investigated the effects of ATF3 on the regulation of bile acid and lipoprotein metabolism in atherosclerosis and demonstrated that hepatocyte ATF3 enhances high-density lipoprotein (HDL) uptake, inhibits intestinal lipid absorption, promotes macrophage reverse cholesterol transport, and ameliorates dyslipidemia. Consistent with these findings, they found that hepatocyte ATF3 regulates HDL metabolism by inducing scavenger receptor group B type 1 (SR-BI) activity via its interaction with p53 and by repressing cholesterol 12α-hydroxylase (CYP8B1) via its interaction with HNF4α. Taken together, these observations indicate that hepatocyte ATF3 plays critical roles in modulating glucolipid metabolism and protecting against metabolic disorders (Figure 2).

Given that the liver is a major site of glucolipid metabolism in the body, targeting hepatic ATF3 may represent a promising approach for the treatment of metabolic diseases caused by hepatic metabolic dysfunction.

## Metabolic functions of ATF3 in the adipose tissue

The adipose tissue, as a widely recognized metabolic and endocrine organ in the body, is integral to the maintenance of glucose and lipid homeostasis through its subtle functions at both the organ and systemic levels. In the classical view, the adipose tissue can be classified into white adipose tissue (WAT), beige adipose tissue, and brown adipose tissue (BAT) according to their visibly distinguishable tissue colors and functions in storing or dissipating energy. Dysregulation of adipocyte differentiation is closely associated with adipose tissue dysfunction, which results in an increased risk of obesity-linked metabolic complications, including insulin resistance, hepatic steatosis, diabetes, and hyperlipidemia (Ghaben and Scherer, 2019; Song and Kuang, 2019). Therefore, regulation of adipocyte differentiation has emerged as a promising strategy for relieving obesity-related metabolic disorders.

A growing number of studies have demonstrated that transcriptional regulation plays a crucial role in adipocyte differentiation (Guo et al., 2015; Lee et al., 2019). It is well known that CCAAT/enhancer-binding protein α (C/EBPα) and peroxisome proliferator-activated receptor-γ (PPARγ) are two master transcription factors for terminal adipocyte differentiation. Interestingly, Ku et al. (2022) recently reported that the ATF3 inducer ST32db can inhibit 3T3-L1 preadipocyte differentiation by inhibiting adipogenesis and enhancing lipolysis via the activation of the β3-adrenoceptor/PKA/p38, AMPK, and ERK pathways. These results are consistent with those of earlier studies showing that lentivirus- or thapsigargin-mediated ATF3 overexpression inhibited adipocyte differentiation by binding to the ATF/CRE site on the promoters of C/EBPα and PPARγ2 and decreasing transcription in 3T3-L1 cells (Jang et al., 2012; Jang and Jung, 2014). Consistent with its function in the inhibition of adipocyte differentiation, ATF3 exerts an anti-obesity effect in mice (Ku et al., 2022). Sulfuretin-induced ATF3 expression in WAT and adipocytes has been found to reduce epididymal WAT and liver weights in mice significantly (Kim et al., 2019). Conversely, global ATF3 deficiency has been found to be associated with increased depot weights in WAT (inguinal WAT, mesenteric WAT, and retroperitoneal WAT) and BAT in mice fed a high-fat diet (Cheng et al., 2019). In view of these studies, ATF3 may be a good therapeutic target for the modulation of adipocyte differentiation to combat obesity.

Mature adipocytes, which are differentiated from preadipocytes, make up most of the volume of the adipose tissue. Much of the research devoted to understanding mature adipocytes in metabolism has indicated that they not only form a reservoir for free fatty acids (FFAs) but also provide an array of adipokines capable of influencing whole-body metabolism (Raajendiran et al., 2016; Unamuno et al., 2018; Kahn et al., 2019). Among adipokines secreted by adipocytes, adiponectin is a well-known homeostatic factor that regulates glucolipid metabolism and insulin sensitivity through its anti-inflammatory, anti-fibrotic, and antioxidant effects. In rodents and humans with obesity or insulin resistance, adiponectin expression markedly decreases (Kadowaki et al., 2006). Interestingly, some evidence shows that ATF3 is a negative regulator of adiponectin. For example, Kim et al. (2006) reported that ATF3 negatively regulates adiponectin gene expression by specifically binding to the putative AP-1 binding site (TGACTCTC, −376/−369) of the adiponectin promoter in 3T3-L1 adipocytes. ATF3 can also transcriptionally repress adiponectin receptor-2 (AdipoR2) gene expression by directly binding to the 5′ flanking promoter region of AdipoR2 and thus interfere with the protective effect of adiponectin on obesity-related insulin resistance (Koh et al., 2010). However, these studies did not provide evidence for anti-obesity effects or the protective role of ATF3 in alleviating obesity-linked metabolic disorders. Therefore, it is important to further clarify the biological functions of ATF3 in different stages of adipocyte development in obesity, which will be more conducive to the development of intervention strategies for metabolic diseases based on targeting ATF3 in the future.

## Metabolic roles of ATF3 in constituent cells of atherosclerotic plaque

Atherosclerosis, a chronic inflammatory disease characterized by lipid deposition and atherosclerotic plaque formation in the intima, is the leading cause of CAD (Shao et al., 2020). Atherosclerotic plaques contain a variety of cells, such as monocyte-derived macrophages, endothelial cells (ECs), leukocytes, and smooth muscle cells (SMCs). The continuous crosstalk between the lipid metabolic imbalance and immune-inflammatory response contributes to the formation of atherosclerotic plaques in the intima (Libby et al., 2011). Here, we summarize the roles of ATF3 in cellular lipid metabolism and the progression of atherosclerosis.

Macrophages play a central role in the pathogenesis of atherosclerosis by participating in both foam cell formation and the inflammatory response (Moore and Tabas, 2011). As a result, macrophages may be targeted for therapy. Emerging studies suggest that ATF3 is a key transcription factor that plays vital roles in modulating the macrophage metabolic state, immunity, and inflammatory responses. Deletion of ATF3 in ApoE^–/–^ mice fed a high-fat diet led to increased foam cell formation and macrophage inflammation during the progression of atherosclerosis by elevating aortic 25-hydroxycholesterol (25-HC) levels. Mechanistically, ATF3 directly binds to the promoter of cholesterol 25-hydroxylase (Ch25h), which leads to epigenetic repression of Ch25h transcription. Ch25h is the key enzyme that catalyzes the production of 25-HC. Histone acetylation at the Ch25h promoter is significantly higher in ATF3^–/–^ than in wild-type (WT) macrophages (Gold et al., 2012). In addition, ATF3 can protect macrophages against C-type lectin receptor 4e (Clec4e) signaling-induced foam cell formation. Loss of ATF3 has been found to aggravate Clec4e-mediated inhibition of cholesterol efflux, as shown by the significant decrease in ATP-binding cassette subfamily A member 1 (Abca1) expression in ATF3^–/–^ macrophages (Clément et al., 2016).

On the other hand, ATF3 is a key regulator of inflammatory responses in macrophages. ATF3 suppresses Toll-like receptor 4 (TLR4)-stimulated inflammatory responses by negatively regulating IL-12p40 promoter activity, leading to the inhibition of foam cell formation in THP-1 macrophages (Whitmore et al., 2007). In addition, macrophages deficient in ATF3 have been found to exacerbate the Clec4e-mediated inflammatory response via increases in Clec4e, Tnfα, and Ccl2 expression (Clément et al., 2016). More importantly, ATF3, as an HDL-inducible transcription factor in macrophages, mediates the protective effects of HDL against TLR-induced inflammation by limiting excessive production of proinflammatory cytokines, including TNFα, IL-6, and IL-12p40, both *in vitro* and *in vivo* (De Nardo et al., 2014). Notably, a gene polymorphism study showed that ATF3 is associated with decreased serum inflammatory marker C-reactive protein levels in non-obese subjects, which can protect against atherosclerotic disease (Wu et al., 2011). The studies described above demonstrate that ATF3 is a key regulator of lipid metabolic and inflammatory pathways in macrophages.

ECs and SMCs are also involved in the pathogenesis of atherosclerosis. In ECs, antisense ATF3/LRF-1 cDNA has been found to partially suppress the cell death induced by TNFα, ox-LDL, and lysophosphatidylcholine, which slows the progression of atherosclerosis (Nawa et al., 2002). However, adenovirus-mediated ATF3 overexpression has been shown to protect human umbilical endothelial cells from TNFα-induced apoptosis through the downregulation of p53 transcription (Kawauchi et al., 2002). Consistent with the findings of the latter study, global ATF3 knockout mice have been found to show elevated serum levels of TNFα and ICAM-1, impaired endothelium-dependent aortic relaxation, and increased aortic wall thickness and lipid peroxides (Chou et al., 2018). In addition, ATF3 is a key repressor of transitioning SMCs toward the subset of cells that promote vascular inflammation by activating the complement cascade (Wang et al., 2021). These mechanistic studies indicate that ATF3 exerts an anti-inflammatory effect and acts as a cell protective factor for ECs and SMCs during atherogenesis.

Taken together, these studies show a protective role of ATF3 in diverse cell types in the vascular wall through the regulation of lipid metabolism and inflammatory responses. ATF3 can be a crucial therapeutic target molecule for the regulation of lipid metabolism and inflammation in the vascular wall to prevent the progression of atherosclerosis.

## Role of ATF3 in glucose and lipid metabolism in the intestine

The intestine is an important metabolic organ that regulates the pathophysiology of various metabolic diseases, including obesity, insulin resistance, and diabetes. Growing evidence has shown that the intestine is linked to systemic glucose and lipid metabolism via regulation of the key aspects of dietary lipid absorption, intracellular transport, and metabolism (Chávez-Talavera et al., 2017; Ticho et al., 2019).

ATF3 is a highly conserved transcription factor that is ubiquitously expressed in *Drosophila*, mice, dogs, monkeys, humans, and so on (Wang et al., 2007). Intestinal ATF3 has been shown to safeguard metabolic and immune system homeostasis in *Drosophila melanogaster*. ATF3 mutation affecting the intestine of larvae or knockdown of ATF3 in the midgut of adult flies has been shown to result in overload of lipids. Mechanistically, ATF3 mutation in *D. melanogaster* promoted the activation of NF-κB/Relish, Jun N-terminal kinase (JNK), and forkhead box-containing protein O (FOXO), resulting in the deregulation of genes important for lipid metabolism and immune defense, such as those encoding lipases (lip3, lip4, CG31089, and CG6271), enzymes involved in triacylglycerol synthesis (wun), and effectors of immune responses (AttA-D, Drs, Dro2, and Dro5) (Rynes et al., 2012).

In addition, the function of intestinal ATF3 has been implicated in stress- and inflammation-induced alterations of lipid metabolism. For example, the role of p38c, which represents one of the stress signaling pathways in the regulation of intestinal lipid homeostasis, is mediated by ATF3 (Chakrabarti et al., 2014). Enterocyte-specific ATF3 inactivation increases JNK signaling activity and intestinal barrier dysfunction-mediated infection. Deficiency of ATF3 leads to chronic inflammation, the fat body phenotype, and elevated counts of natural commensal populations (*Acetobacter* spp.) in the larval gut epithelium. The condition is linked to JNK-mediated cellular stress and inflammation reactions, as well as FOXO activation-related metabolic imbalances (Zhou et al., 2017).

Collectively, ATF3 is essential for protecting intestinal cells from lipid disorders, oxidative stress, and inflammation. However, the role of intestinal ATF3 in glucolipid metabolism in mammals remains unknown and needs to be explored in the future.

## Role of ATF3 in glucose and lipid metabolism in other metabolic organs

The pancreas, hypothalamus, and muscle are also critical for modulating glucose, insulin, lipid, and energy metabolism. With regard to the pancreas, there are major discrepancies among previous reports on the physiological role of ATF3 in the regulation of glucose metabolism. Both pancreatic ATF3 and pancreatic β cell-specific ATF3 transgenic mice have been found to show decreased β cell mass and severe glucose intolerance, displaying a deleterious function of ATF3 (Kim et al., 2010). However, compared with control mice, conventional ATF3 knockout mice have been shown to develop more severe glucose intolerance when fed a high-fat diet due to transcriptional repression of insulin gene expression (Zmuda et al., 2010a), indicating that ATF3 has a beneficial role in the pancreas. Such contradictory results have led researchers to perform additional studies to elucidate the physiological role of pancreatic ATF3 in glucose metabolism.

Lee et al. (2014) found that ATF3 expression is upregulated by low glucose in pancreatic α and β cells, resulting in increased transcription of the glucagon gene but not the insulin gene. Zmuda et al. (2010b) found that ATF3 deficiency in islets protected them from stress stimulation, including islet isolation, inflammatory cytokine treatment, and hypoxia. The islets of ATF3 knockout mice displayed improved glucose homeostasis compared with those of WT mice after islet transplantation. Mechanistically, ATF3 knockout islet grafts showed decreased levels of the proapoptotic genes Noxa and Bnip3, lower expression of the inflammation-related genes Tnfα, IL-1β, and IL-6, and reduced caspase-3 activation and macrophage infiltration. These observations show a deleterious function of pancreatic ATF3 in glucose metabolism resulting from upregulation of peptide hormone genes, apoptosis-related genes, and inflammation-related genes by ATF3.

In addition, Lee et al. (2013) reported that hypothalamic ATF3 knockout mice exhibited improved glucose tolerance, while the plasma levels of glucagon and insulin were unchanged. Interestingly, loss of hypothalamic ATF3 in mice resulted in a leaner phenotype, decreased food intake, and enhanced energy expenditure as a result of ATF3 interacting with FOXO1 at the agouti-related protein (Agrp) promoter to upregulate Agrp transcription. To date, the metabolic functions of ATF3 in muscle tissues remain unknown, although some studies have shown that ATF3 is highly expressed and regulates chemokine expression in muscle cells (Fernández-Verdejo et al., 2017; Park et al., 2021). Taken together, these studies suggest that ATF3 plays an important role in regulating glucose metabolism in various metabolic organs and tissues. It is noteworthy that the role of ATF3 in glucose homeostasis remains to be further elucidated in various organs and tissues.

## Function of ATF3 in inter-organ crosstalk in metabolic disorders

**Figure 3:**
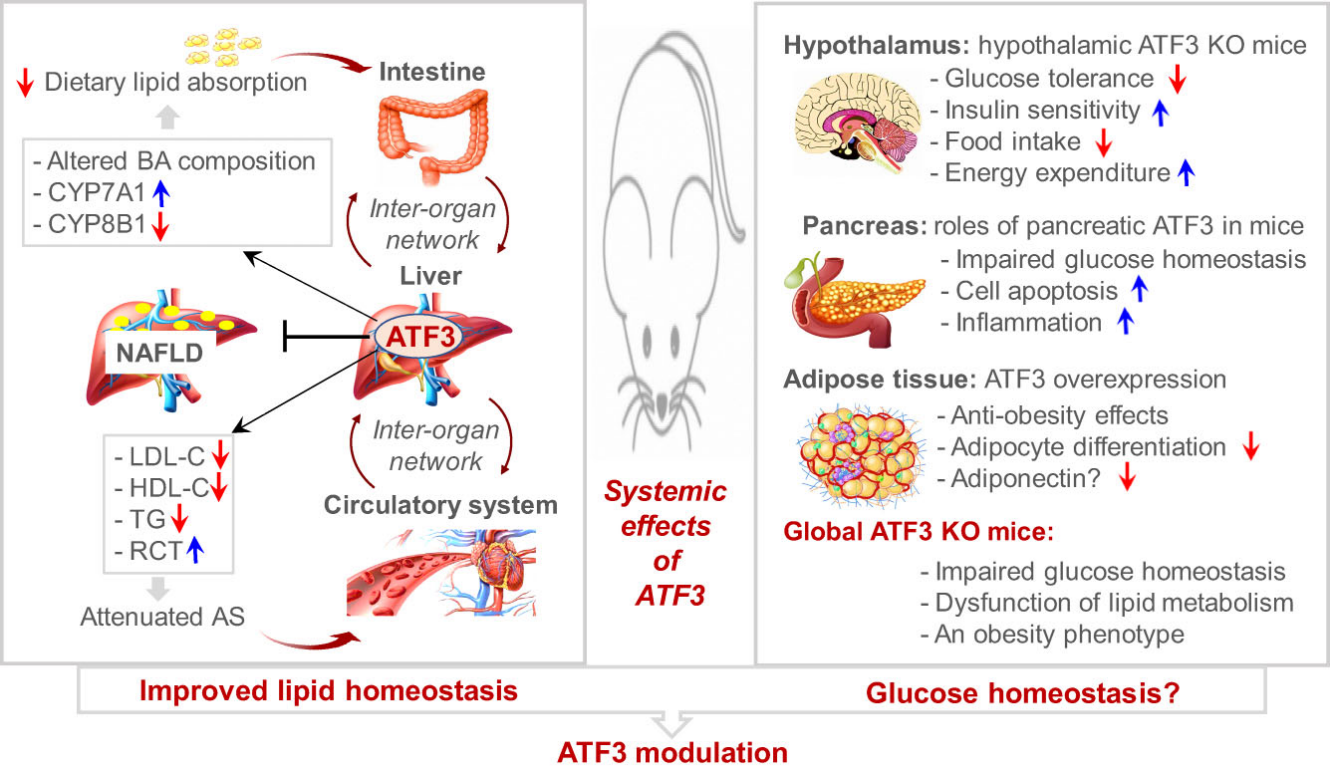
Systemic effects of ATF3 on glucolipid metabolism in mice. Left: ATF3 plays an important protective role in lipid metabolism, especially in the liver. Hepatic ATF3 prevents the progression of NAFLD and atherosclerosis (AS) through regulation of the inter-organ network among the liver, intestine, and circulatory system. Right: the functions of ATF3 in different organs are largely different. Global ATF3 knockout (KO) mice display impaired glucose homeostasis. The role of ATF3 in glucose metabolism needs to be further explored. BA, bile acid; LDL-C, low-density lipoprotein cholesterol; RCT, reverse cholesterol transport.

Maintaining normal life activities in higher organisms requires coordination and unity among organs. Cumulative evidence has demonstrated that continuous and dynamic inter-organ crosstalk is closely associated with the development and progression of metabolic disorders (Castillo-Armengol et al., 2019). Communication among organ systems is controlled mainly by the autonomic nervous system, the endocrine system, and the circulatory system. Studies from our group and others have provided a new perspective on ATF3 participation in crosstalk communication among multiple organs through the regulation of cytokine expression and secretion, lipoprotein metabolism, and bile acid metabolism. Hepatocyte ATF3 slows the atherosclerotic process in the arterial wall via the regulation of lipoprotein metabolism in the circulatory system and intestinal lipid absorption (Xu et al., 2021b). In addition, adipocyte ATF3 can also influence glucolipid metabolism-related metabolic disorders by controlling adiponectin expression and secretion (Favre et al., 2011). Therefore, ATF3 is a key transcription factor that modulates glucolipid metabolism and related metabolic diseases by controlling a metabolic and inflammatory network through specific organs and cell types and inter-organ crosstalk (Figure 3). Although the role of ATF3 in glucose homeostasis remains to be fully determined, global ATF3 inactivation in mice harms glucose metabolism. Thus, modulation of ATF3 activity holds the potential to improve glucolipid metabolic disorders and related metabolic diseases.

## Potential regulators of ATF3 in metabolic disease

**Figure 4:**
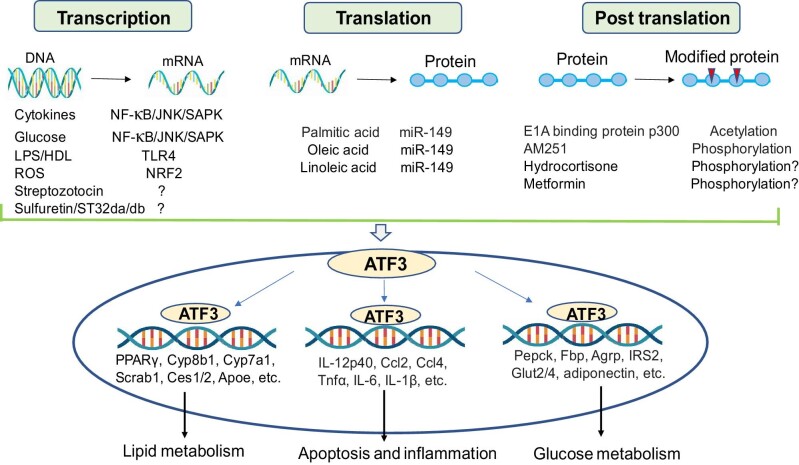
Potential regulators of ATF3 that have been implicated in glucolipid metabolism and inflammation. A variety of factors such as cytokines, nutrients, LPS, ROS, and hormones can trigger several intracellular signaling cascades, subsequently regulating the expression of ATF3 in cells. ATF3 protein has various functions by activating/repressing its target genes under physiological and pathological conditions in metabolic diseases. ATF3 can modulate the expression levels of lipid metabolism-related genes (PPARγ, Cyp8b1, Cyp7a1, Scrab1, Ces1/2, and Apoe), glucose metabolism-related genes (Pepck, Fbp, Agrp, IRS2, Glut2/4, and adiponectin), and inflammation-related genes (IL-12p40, Ccl2, Ccl4, Tnfα, IL-6, and IL-1β) via binding to their promoters.

ATF3 is a highly regulated transcription factor that is implicated in a wide range of vital physiological processes, including lipid metabolism, glucose metabolism, apoptosis, and inflammation. ATF3 is regulated at multiple levels, including regulation at the level of transcription initiation by signal transduction cascades and transcription factors, regulation at the translation level by miR-149, which regulates mRNA stability and translation by binding to noncoding regions of ATF3, and regulation at the posttranslational level by protein modifications (Figure 4).

ATF3 activity is induced in response to oxidative stress conditions, inflammatory responses, cytokines, glucose, and HDL. For example, ATF3 induction by proinflammatory cytokines and glucose is partially mediated by the NF-κB and JNK/stress-activated protein kinase (SAPK) signaling pathways, which are two stress-induced pathways involved in inflammatory responses and diabetes (Hartman et al., 2004). ATF3 is also upregulated by TLR4 signaling (LPS/HDL) and participates in the negative feedback loop that modulates the inflammatory response (Whitmore et al., 2007; De Nardo et al., 2014). Reactive oxygen species (ROS)-mediated superinduction of ATF3 is partially mediated by NF-E2-related factor 2 (NRF2) (Hoetzenecker et al., 2011). Streptozotocin treatment upregulates the expression of ATF3 in diabetic liver injury. In addition, although researchers have found that ATF3 transcription can be upregulated by some compounds, such as sulfuretin and ST32da/db (Cheng et al., 2019; Kim et al., 2019; Ku et al., 2022), the detailed mechanisms remain unknown. On the other hand, ATF3 abundance can be regulated at the translational and posttranslational levels. Hepatic ATF3 can be repressed by miR-149, which is markedly upregulated by nutritional signaling compounds, including palmitic acid (PA), oleic acid (OA), and linoleic acid (LA). miR-149 is induced in NAFLD, and the inhibition of ATF3 expression in NAFLD may be mediated, at least in part, by the induction of hepatocyte miR-149 (Xu et al., 2021a). ATF3 has also been shown to be modified by posttranslational modifications such as acetylation, phosphorylation, and SUMOylation. E1A-binding protein p300-mediated ATF3 acetylation is crucial for ATF3 binding to Gadd45β/γ promoters and consequently enhances Gadd45β/γ transcription and cell apoptosis (He et al., 2016). Furthermore, AM251, a cannabinoid antagonist, has been exhibited to increase the phosphorylation of ATF3, resulting in inhibition of the viability of hepatoma HepG2 cells (Lee et al., 2008). Moreover, hydrocortisone represses hepatic ATF3 activity by activating the cAMP–protein kinase A pathway (Xu et al., 2021b), whereas metformin can induce ATF3 expression by activating AMPK signaling (Luizon et al., 2016). Although the detailed mechanisms have not been determined, phosphorylation is thought to exert important roles in the regulation of ATF3 expression by hydrocortisone and metformin.

ATF3 is critical for the regulation of glucolipid metabolism and inflammatory response via activation/repression of its target genes, such as lipid metabolism-related genes (PPARγ, Cyp8b1, Cyp7a1, Scrab1, Ces1/2, and Apoe), glucose metabolism-related genes (Pepck, Fbp, Agrp, IRS2, Glut2/4, and adiponectin), and inflammation-related genes (IL-12p40, Ccl2, Ccl4, Tnfα, IL-6, and IL-1β) (Figure 4). Therefore, induction of ATF3 can protect against the development and progression of metabolic disease, and a more detailed understanding of approaches for regulating ATF3 activity would make ATF3 a promising target not only for glucolipid metabolic disorder therapy but also for multidisciplinary metabolic disease therapy.

## Concluding remarks and perspectives

ATF3, as an adaptive-response gene, is widely expressed in various cells and inhibits inflammatory responses, tumorigenesis, and cancer progression. Emerging evidence shows that ATF3 can also modulate glucose and lipid metabolism by sensing extracellular nutritional signals and regulating the transcription of genes involved in glucose and lipid metabolism, thereby playing an essential physiological and pathophysiological role in the development of metabolic disorders. Collectively, these findings indicate that ATF3 acts as a novel regulator of glucose and lipid disorders, the two leading causes of metabolic-related diseases. Thus, ATF3 is a very promising and valuable therapeutic target. However, there are several challenges in developing ATF3-based therapies.

First, the pathophysiological function of ATF3 in glucose metabolism appears to be tissue/cell type-dependent. Hepatic ATF3 alleviates gluconeogenesis by inhibiting the transcription of PEPCK and FBP, two rate-limiting enzymes involved in gluconeogenesis (Allen-Jennings et al., 2001). However, transgenesis of mice to influence pancreatic ATF3 expression has been found to worsen glucose intolerance by decreasing β cell mass (Kim et al., 2010). Thus, cell type/tissue-specific manipulation of ATF3 expression is essential for therapeutic intervention to avoid unwanted side effects. A better understanding of how ATF3 regulates signaling pathways involved in glucose metabolism in various cell types or tissues is warranted in the future.

Second, ATF3 does not function alone in metabolic disease and is known to cooperate with transcription factors, as well as other regulatory factors associated with glucolipid metabolism and inflammation. The relationship between ATF3 and HNF4α is of particular interest because both are inactivated by a remarkably common set of metabolic stresses triggered by FFAs. Furthermore, both transcription factors play essential roles in glucolipid metabolism. In addition, the interactions of ATF3 with NF-κB and p53 appear to affect the expression of proinflammatory cytokines and apoptotic genes. ATF3 acts as a metabolic stress-sensitive transcription factor and halts the development of metabolic diseases, including inflammation-linked glucolipid metabolic disorders. Precisely how ATF3 cooperates with other regulatory factors is an important question to be addressed.

In summary, ATF3 is an important regulator of lipid, bile acid, HDL, and glucose metabolism and inflammation. Increased ATF3 activity exerts beneficial effects on obesity, fatty liver disease, and atherosclerosis. ATF3 may be a therapeutic target for the treatment of these metabolic disorders.
